# Metabolic profiles in five high-producing Swedish dairy herds with a history of abomasal displacement and ketosis

**DOI:** 10.1186/1751-0147-50-31

**Published:** 2008-08-07

**Authors:** Lena Stengärde, Madeleine Tråvén, Ulf Emanuelson, Kjell Holtenius, Jan Hultgren, Rauni Niskanen

**Affiliations:** 1 Division of Ruminant Medicine and Epidemiology, Department of Clinical Sciences, Swedish University of Agricultural Sciences, P.O. Box 7054, SE-75007, Uppsala, Sweden; 2 Department of Animal Nutrition and Management, Swedish University of Agricultural Sciences, Kungsängen Research Centre, SE-75323, Uppsala, Sweden; 3 Department of Animal Environment and Health, Swedish University of Agricultural Sciences, P.O. Box 234, SE-53223, Skara, Sweden; 4 Meat Control Division, National Food Administration, P.O. Box 622, SE-75126, Uppsala, Sweden

## Abstract

**Background:**

Body condition score and blood profiles have been used to monitor management and herd health in dairy cows. The aim of this study was to examine BCS and extended metabolic profiles, reflecting both energy metabolism and liver status around calving in high-producing herds with a high incidence of abomasal displacement and ketosis and to evaluate if such profiles can be used at herd level to pinpoint specific herd problems.

**Methods:**

Body condition score and metabolic profiles around calving in five high-producing herds with high incidences of abomasal displacement and ketosis were assessed using linear mixed models (94 cows, 326 examinations). Cows were examined and blood sampled every three weeks from four weeks ante partum (ap) to nine weeks postpartum (pp). Blood parameters studied were glucose, fructosamine, non-esterified fatty acids (NEFA), insulin, β-hydroxybutyrate, aspartate aminotransferase, glutamate dehydrogenase, haptoglobin and cholesterol.

**Results:**

All herds had overconditioned dry cows that lost body condition substantially the first 4–6 weeks pp. Two herds had elevated levels of NEFA ap and three herds had elevated levels pp. One herd had low levels of insulin ap and low levels of cholesterol pp. Haptoglobin was detected pp in all herds and its usefulness is discussed.

**Conclusion:**

NEFA was the parameter that most closely reflected the body condition losses while these losses were not seen in glucose and fructosamine levels. Insulin and cholesterol were potentially useful in herd profiles but need further investigation. Increased glutamate dehydrogenase suggested liver cell damage in all herds.

## Background

At the onset of lactation the nutrient demand increases dramatically and faster than the increase in feed intake. Thus most dairy cows face negative energy balance (NEB) in early lactation. Postpartum (pp) feed intake is lower in cows with higher body condition scores (BCS) ante partum (ap), leaving them in NEB for a longer period than cows with normal or low BCS [1,2]. Most diseases in dairy cows occur during the first two weeks pp [3]. Metabolic disorders are highly multi-factorial and a wide range of animal, management and feed factors may lead to such problems. Fatty liver may occur around calving when the cow is in NEB and blood levels of non-esterified fatty acids (NEFA) increase as the cow mobilizes adipose tissue. Fatty liver has been shown to be associated with other diseases in the periparturient period [4].

Blood profiles have frequently been used to assess nutrient status of cows in the transition period [5-9]. Also the BCS is used in the management of dairy herds [10]. Early blood profiles included packed cell volume and haemoglobin [6] along with glucose, proteins and minerals. More recently, metabolites such as NEFA and β-hydroxybutyrate (BHB) have been added to the profiles to monitor energy balance. Blood profiles are considered useful to identify nutritional shortcomings even before the productivity is impaired [11]. Such profiles have also been used to monitor herd health and to find subclinical disease, to predict risk of ketosis or abomasal displacement as well as investigate herd problems with metabolic disorders [12-15].

Blood parameters that may reflect nutrient status of the cow, such as glucose, fructosamine, insulin, NEFA, BHB and cholesterol, also enzymes and proteins that reveal liver status are of interest to include in transition period profiles. Fructosamines are complexes produced by an irreversible, nonenzymatic glycosylation of proteins and serum concentrations depend on glucose and protein concentrations and provide a retrospective record of blood glucose levels during the previous one to three weeks. Fructosamine has been suggested as a parameter to monitor glucose levels over longer periods in an attempt to avoid the variability in glucose associated with diurnal fluctuations [16-18]. In accordance with observations in companion animals and humans where fructosamine is used to monitor blood glucose levels in diabetic patients [19,20], cows with markedly elevated levels of glucose have been observed to have elevated levels of fructosamine [Tråvén and Holtenius, unpublished data].

Part of the variation in cholesterol may be explained by dry matter intake [21], where a lower feed intake leads to lower cholesterol levels. Low cholesterol levels the first weeks after calving have also been associated with fatty liver pp [22-24]. The liver cell enzymes aspartate aminotransferase (AST) and glutamate dehydrogenase (GD) may leak into the blood stream when liver cell damage occurs in dairy cattle [25]. The acute phase protein haptoglobin rises in response to inflammation [26]. It has also been associated with fatty liver in dairy cows [27,28]. It is therefore of interest to study an extended palette of blood parameters where new candidates such as fructosamine, haptoglobin, GD and cholesterol may be introduced in herd health management.

The aim of this study was to examine BCS and extended metabolic profiles, reflecting both energy metabolism and liver status around calving in high-producing herds with a high incidence of abomasal displacement and ketosis and to evaluate if such profiles can be used at herd level to pinpoint specific herd problems.

## Methods

Dairy herds enrolled in the Swedish official milk recording scheme (SOMRS) with more than 100 cows and a production of at least 9 500 kg energy-corrected milk (ECM) per cow annually (representing 85%, 7.8% and 38% of Swedish dairy herds, respectively) were eligible for inclusion in this study. Abomasal displacement and ketosis were chosen as indicators of metabolic imbalances in the transition period. To find long-term problem herds, practicing veterinarians in two regions, around the Skara and Uppsala areas, were asked for eligible herds where abomasal displacement and ketosis had been a problem for several years. The herds had to have a minimum of 6 cases of abomasal displacement or ketosis or both per 100 lactating cows within the last year to be identified as herds with a high disease frequency compared to the Swedish average herd. The average incidence of abomasal displacement and ketosis in 2005/2006 was 1.0 and 1.3 cases per 100 cows, respectively [29]. The sample size was set to five and the first five herds that were asked to participate accepted (herds A-E).

Herds A, B, C and E were visited during the period of January-June and herd D and E were visited during September-December 2005. All herd visits were carried out by one veterinarian (LS), except for three consecutive visits in herd E that were carried out by another veterinarian. At each visit, dry cows and heifers within four weeks of expected calving were clinically examined and blood was sampled. The cows were re-examined and re-sampled every three weeks until nine weeks pp and until at least 15 cows had been examined in each herd. The clinical examination included general condition and BCS. BCS was assessed on a five-point scale with half-point increments, where one represents an emaciated cow and five a severely overconditioned cow [30]. BCS ap was defined as BCS four weeks to one day ap. If cows were scored twice ap, the averages of the scorings were used. BCS loss was defined as the difference between BCS ap and BCS at week 4–6 pp.

In each herd, information about management, housing, feed and herd health was gathered through standardized questions by the visiting veterinarian. Herd data on milk yield, herd size and disease incidence, as well as individual data for cows, such as breed, date of calving, parity and diseases during the study period, were retrieved from the SOMRS. The study, including the handling of animals, was approved by the local ethics committee in Göteborg, Sweden (reference number 269–2004).

### Feed hygiene samples

Samples of silage and grain produced on-farm were taken at storage in silos or in the grain roller. The samples were analysed for hygiene parameters with commercial methods at the laboratory of the Department of Animal Feed, National Veterinary Institute, Uppsala, Sweden. Silage samples were analysed for pH and microbial growth and grain samples were analysed for water activity, microbial growth, and per cent endogenously infected kernels. The microbiological quality of the feed samples was assessed by the analysing laboratory.

### Blood samples

Blood from the coccygeal vein or artery of each cow was collected in evacuated test tubes without additives and tubes with FluoridHeparin (CAT/FH, BD Vacutainer Systems). Blood samples were refrigerated and transported to a field station. Samples were centrifuged at 2000 g for 10 min and serum and plasma were harvested, and frozen within 6 h from sampling. Samples were stored at -20°C up to 5 months before analysis.

Serum and plasma samples were analysed at the Clinical Pathology Laboratory, University Animal Hospital, SLU, Uppsala using commercial kits according to the manufacturers' instructions. Haemolysed samples were excluded (n = 1). Serum haptoglobin (PHASE RANGE, Haptoglobin Assay, Tridelta Development Ltd, Bray, Ireland) and BHB (β-Hydroxybutyrate LiquiColor Procedure No. 2440, STANBIO laboratory, Boerne, TX., US) were analysed on a Cobas MIRA chemistry analyser (Roche Diagnostica, Basel, Switzerland). Serum activities of AST (AST/GOT, IFCC, Konelab, Thermo Electron Corporation, Vantaa, Finland), GD (GLDH, Roche Diagnostics GmbH, Mannheim, Germany), as well as concentrations of total cholesterol(CHOLESTEROL, Konelab, Thermo Electron Corporation), NEFA (NEFA C, ACS-ACOD method, Wako Chemicals GmbH, Neuss, Germany), fructosamine (Fructosamine, ABX Pentra, Montpellier, France) and plasma glucose (GLUCOSE, HK, Konelab, Thermo Electron Corporation) were determined on a Konelab 30 chemistry analyser (Thermo Electron Corporation). Serum insulin was analysed with a porcine insulin radioimmunoassay (PORCINE INSULIN RIA KIT, Linco research, St. Charles, MO., US) using a Cobra II Auto-Gamma counter (Packard Instrument Company, Meriden, CT., US).

### Data analyses

Cows with only one observation (39 cows), and observations from cows with a disturbed general condition at clinical examination or diagnosed with clinical disease ± 5 days from sampling (18 observations) were excluded. Further, observations ± 1 day around parturition were excluded from the analyses (17 observations) because blood metabolites during this period are more affected by parturition per se and may thus be less useful as indicators of metabolic status. The remaining 326 observations were included in the study.

Herd-specific patterns for blood parameters and BCS were analysed by linear mixed models as applied in the MIXED procedure of SAS (SAS version 9.1, SAS Institute, Cary, NC., USA). The repeated statement and an unstructured covariance matrix were used to account for the repeated sampling within cow and herd. All outcome variables were measured on a continuous scale. BCS, glucose and fructosamine were used without transformations while NEFA, insulin, BHB, AST, GD and haptoglobin were log-transformed and cholesterol was square-root-transformed to get normally distributed residuals. Predictor variables included as fixed effects in the models were breed, parity, week, herd and the interaction between week and herd. Breed was classified into Swedish Red, Swedish Holstein and other/crossbreeds. Parity was classified as first, second or third or more lactations. Week was classified as one-week intervals from 3 weeks ap to 9 weeks pp, but values from week 4 ap were included in week 3 ap.

The statistical significance of differences in herd-specific patterns was tested by combining weekly estimates from the models in four periods using the estimate statement in the MIXED procedure; four weeks ap to partum, partum to three weeks pp, 4–6 weeks pp and 7–9 weeks pp. Model validation was done by examining residuals with respect to equal variance and a normal distribution, and all models were found to be valid. A coefficient of determination (R^2^) was approximated by the squared correlation between observed and predicted values [31].

## Results

### Herd data

Ninety-four clinically healthy cows and a total of 326 examinations were included in the study. The herds had 150 to 300 cows per herd and produced 9 500 to 11 500 kg ECM per cow and year. The distribution of cows and observations over predictor variables is shown in Table 1. Cows were milked twice daily except for high-yielding cows in herd B, that were milked in an automated milking system (average number of milkings was 2.5 times/day). All herds kept lactating cows in loose housing systems where they were fed total mixed rations. Herd A had a concentrate:roughage ratio of 55:45 and top fed cows according to milk production. Herd B had a ratio of 30:70, herd C a ratio of 30:70, herd D a ratio of 60:40 and herd E a ratio of 60:40. Dry cows received straw ad lib and a restricted ration of either TMR or silage. Feed to dry cows in herd A consisted of only straw one day and restricted rations of TMR the next day until close-up diet was applied 2–3 weeks ap. As close-up diets, all herds received restricted rations of TMR starting 2–3 weeks ap. In week 6 pp, herd D changed to a batch of silage (according to the questionnaire) in which hygiene quality problems were detected. Herds A and E held dry cows in tie stalls and herds A, B and C held dry cows and heifers separate from lactating cows until after calving. Herd A and B had a separate group for dry animals receiving close-up diet while herd C held all dry cows and heifers in one group irrespective of time to calving. Disease frequency and recorded cases of abomasal displacement and ketosis per 100 cows in the 12-month period preceding the last herd visit are shown in Table 2.

**Table 1:** Distribution of observations over class predictor variables

Predictor variable	Class	Number of cows	Number of samples
Breed	Swedish Red	12	41
	Swedish Holstein	56	192
	Other breeds or crossbreeds	26	93
Parity	First lactation	19	67
	Second lactation	35	128
	Third or more lactations	40	131
Week	w 4 and 3 ap		38
	w 2 ap		28
	w 1 ap		28
	w 1 pp		19
	w 2 pp		22
	w 3 pp		37
	w 4 pp		33
	w 5 pp		24
	w 6 pp		32
	w 7 pp		30
	w 8 pp		20
	w 9 pp		15
Herd	A	19	69
	B	22	74
	C	18	59
	D	13	42
	E	22	82

antepartum (ap), postpartum (pp)

**Table 2:** Disease frequency in number of cases (and per cent) in herds A-E during the 12-month period preceding the last herd visit

Herd	Cow-years	Date of last visit	DA	K	DA+K	Total
A	149	2005-06-14	6 (4.0%)	8 (5.4%)	9.4%	97 (65.1%)
B	263	2005-05-02	6 (2.3%)	4 (1.5%)	3.8%	39 (14.8%)
C	499	2005-06-15	8 (1.6%)	7 (1.4%)	3.0%	181 (36.3%)
D	151	2005-12-08	9 (6.0%)	1 (0.6%)	6.6%	70 (46.4%)
E	164	2005-12-22	9 (5.5%)	1 (0.6%)	6.1%	141 (86.0%)

displaced abomasum (DA), ketosis (K) and total disease incidence (Total) recorded

### Feed hygiene

The pH in silage samples ranged from 2.8 to 4.2. Single silage samples from herds A, B, C and the first sample in herd D and E had acceptable microbiological quality. Reduced microbiological quality was found in silage samples from herd D and E. Silage from herd D was sampled again after a change from first cut to second cut, due to diarrhoea in all cows, and detectable levels of *Enterobacteriaceae *and abundant growth of *Aspergillus fumigatus *was found. In the first two silage samples from herd E during the fall, detectable and abundant growth of *Penicillium roqueforti *was found, respectively. The third sample held acceptable microbiological quality.

Herds A, B, D and E used grain produced on-farm. The water activities in the samples were from 0.69 to 0.76. Herds A and B had grain samples with reduced microbiological quality. Grain from herd A had an elevated water activity of 0.71 (reference value < 0.70 [32]), sparse growth of *Penicillium *spp., moderate growth of *Aspergillus fumigatus *and abundant growth of *Fusarium *spp. and *Cladosporium *spp. Grain from herd B had an elevated water activity of 0.76. Aerobic bacteria (log 8.5) and moulds (log 5.3) were considered above threshold (<log 7.7 and <log 5.0, respectively [33]), and 20% of the kernels were endogenously infected with *Fusarium *spp.

### BCS and blood parameters

**Figure 1:**
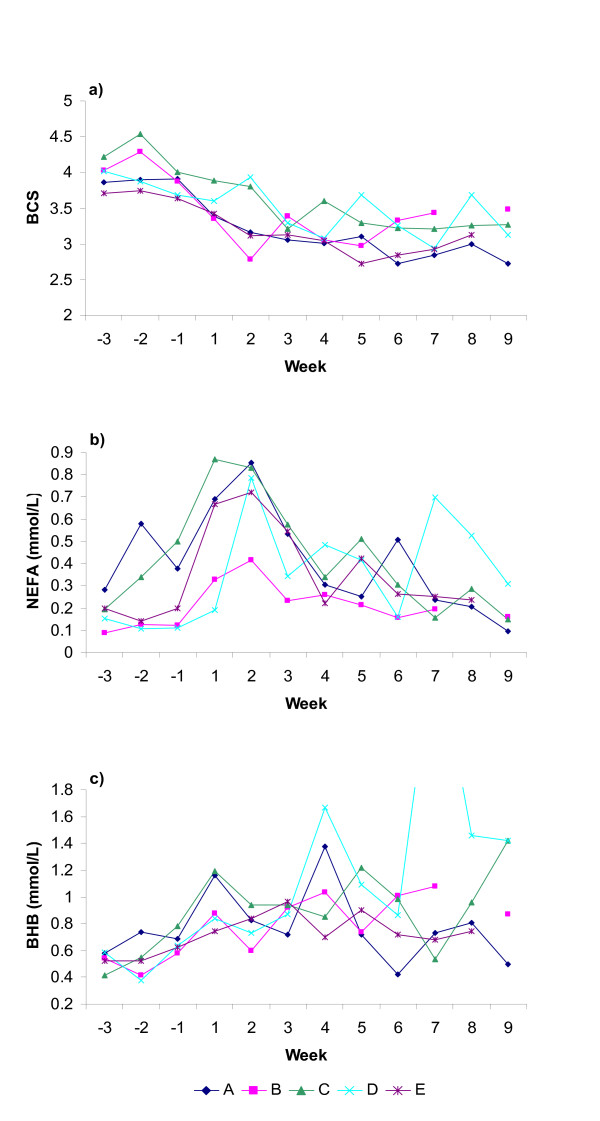
**Herd-specific patterns for herd A-E shown as least-squares means**. a) body condition score (BCS), b) non-esterified fatty acids (NEFA) and c) β-hydroxybutyrate (BHB). The BHB peak week 7 in herd D was 2.95 mmol/L.

**Figure 2:**
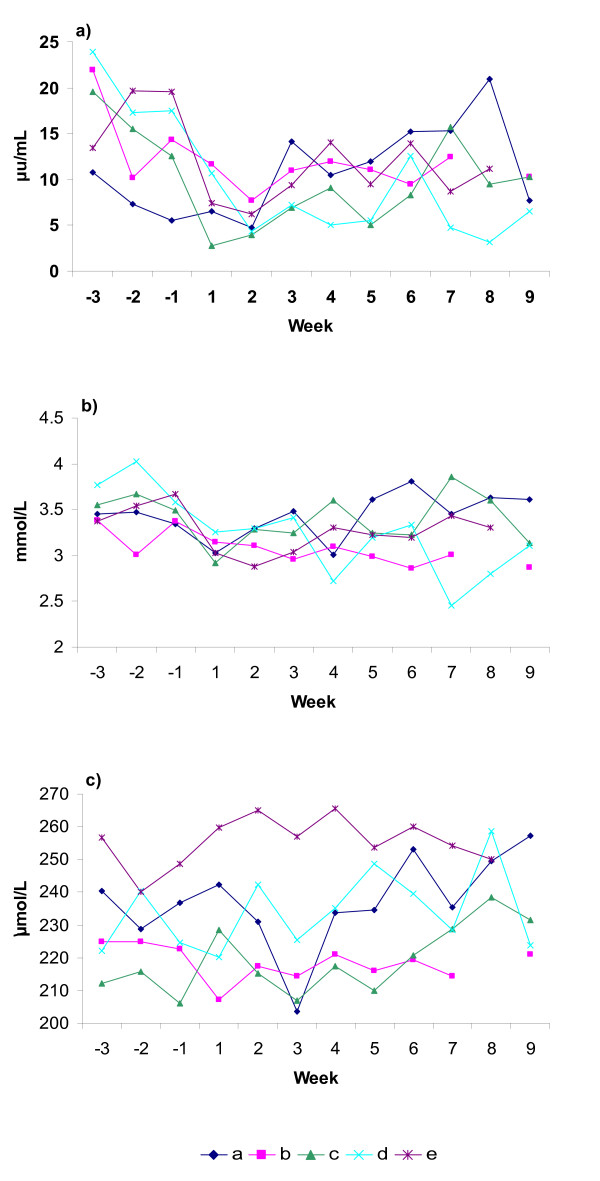
**Herd-specific patterns for herd A-E shown as least-squares means**. a) insulin, b) glucose and c) fructosamine.

**Figure 3:**
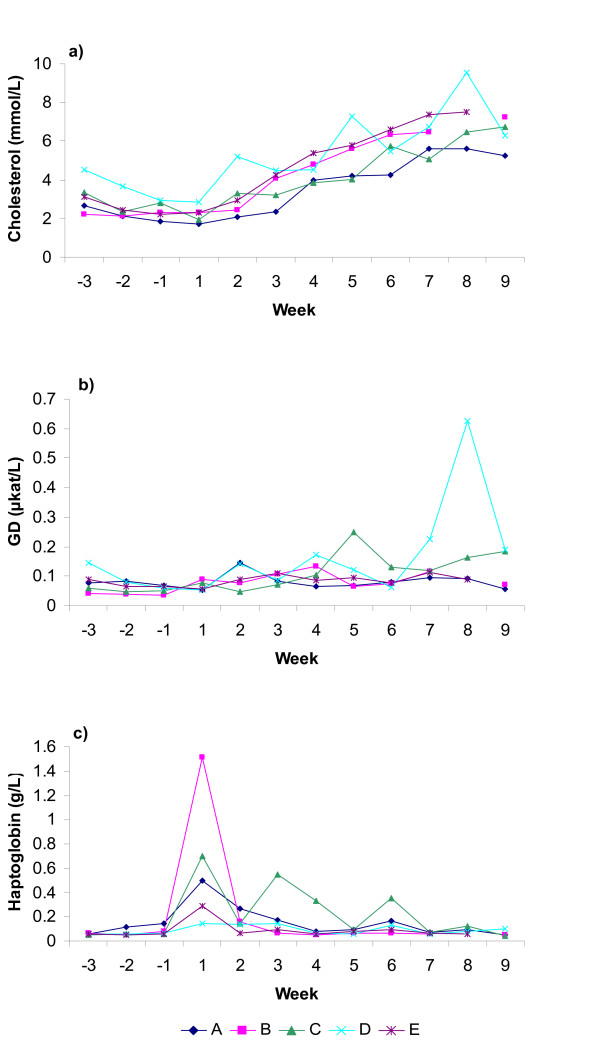
**Herd-specific patterns for herd A-E shown as least-squares means**. a) cholesterol, b) glutamate dehydrogenase (GD) and c) haptoglobin.

Herd- and week-specific estimates (least-squares means) from the linear regression models for BCS, NEFA, BHB, cholesterol, glucose, fructosamine, insulin, haptoglobin and GD are shown in Figures 1, 2, 3. The approximated R^2 ^indicated that the models explained between 37 and 85% of the total variability. The models of haptoglobin, GD and glucose had the lowest R^2 ^and cholesterol had the highest R^2^.

All herds had a mean BCS above 3.5 ap (Fig. 1a). Only 11% of the cows had a BCS of 3 or lower ap, mainly found in herds B and E. All the cows with a BCS loss of 1.0 or greater had a BCS of 4 or higher ap except for one cow each in herds A and E (Table 3). Herds A and C had significantly higher NEFA values than the other herds ap (p < 0.01) and herd B had a significantly lower peak pp than herds A, C and E (p < 0.01, Fig. 1b). Herd A had significantly lower insulin levels ap than the other herds (p < 0.01, Fig. 2a) as well as significantly lower cholesterol levels during the first three weeks pp than the other herds (p < 0.01, Fig. 3a). All individual AST values were below the suggested reference value, <2.2 μkat/L [25], except 1 ap and 6 pp samples. Herd C had significantly higher haptoglobin level than herds D and E and herd A significantly higher level than herd E during the first three weeks pp (p < 0.05). Number of cows and per cent of cows in herds A-E outside suggested reference ranges are shown in Table 3.

**Table 3:** Number of cows (and per cent) in herds A-E outside suggested reference ranges out of those sampled within the time frames

	Herd				
	A (%)	B (%)	C (%)	D (%)	E (%)
NEFA ≥0.4 mmol/L^a ^day 14 to 2 ap^b^	11/13 (85)	0/14 (0)	6/11 (55)	0/8 (0)	1/13 (8)
NEFA ≥0.7 mmol/L day 10 to 20 pp^c^	7/10 (70)	0/13 (0)	3/6 (50)	1/4 (25)	6/18 (33)
BHB ≥1.4 mmol/L day 5–50 pp^d^	7/46 (15)	7/50 (14)	4/38 (11)	7/21 (33)	5/55 (9)
BCS^e ^≥3.5 ap	18/18 (100)	18/21 (86)	13/13 (100)	10/11 (91)	13/20 (65)
BCS loss^f ^>1	6/16 (38)	6/17 (35)	3/12 (25)	1/8 (13)	5/17 (29)
Cholesterol <2.0 mmol/L^g ^day 2–21 pp	7/23 (30)	1/20 (5)	1/13 (8)	0/11 (0)	0/26 (0)
GD >0.25 μkat/L^h ^day 2–21 pp	4/23 (17)	3/20 (15)	1/13 (8)	2/11 (18)	2/26 (8)
Haptoglobin >0.5 g/L^i ^day 2–21 pp	10/23 (44)	2/20 (10)	5/13 (39)	3/11 (27)	5/26 (19)

non-esterified fatty acids (NEFA), β-hydroxybutyrate (BHB), body condition score (BCS), glutamate dehydrogenase (GD), antepartum (ap), postpartum (pp)

^a^Reference range according to Whitaker [11]

^b^Timespan according to Oetzel [12]

^c^Reference range and timespan according to Whitaker [11]

^d^Reference range and timespan according to Oetzel [12]

^e^Edmonson et al. [30]

^f^BCS loss defined as difference in BCS 4 weeks to 1 day ap to BCS at 4–6 weeks pp.

^g^Holtenius et al. [24] and van den Top et al. [22]

^h^Reference according to Clinical Pathology Laboratory, University Animal Hospital, SLU, Uppsala [Personal communication B. Jones]

^i^Reference value chosen to allow for increase in haptoglobin associated with calving.

## Discussion

All herds in the present study had generally overconditioned dry cows. Several studies have shown that overconditioned dry cows have a greater depression of feed intake ap and pp and deeper negative energy balance than cows with a lower body condition [1,2]. In the present study 13% to 38% of the cows in all herds lost over 1.0 unit in BCS in early lactation, up to six weeks pp (Table 3). High BCS ap, as well as major losses in body condition have been associated with abomasal displacement, ketosis and other metabolism related diseases, decreased fertility and increased culling rates [2,34,35]. The high BCS ap and loss of BCS pp in all 5 herds most likely were major contributing factors to the herd problems with metabolic disorders.

Assessing the metabolic blood profiles may aid in investigating the herd problems by indicating the severity and timing of disturbed energy metabolism. Thus herds A and C had higher levels of NEFA ap than the other herds, for herd C mainly during the last week ap. A majority of the cows in these herds had NEFA levels ap above the reference value of 0.4 mmol/L used by Whitaker [11] (Table 3), indicating a mobilisation of adipose tissue already before calving. Cows in herd A also had lower insulin levels ap than the other herds. It has previously been shown that the level of insulin is related to nutrient intake in dry cows [36]. It is thus reasonable to assume that the low insulin level among cows in herd A reflected a low energy intake. The dry cow feeding regime in herd A (according to the questionnaire) using forage with very low energy content during most of the dry period may have caused underfeeding, thus explaining the metabolic profiles. Cows in late pregnancy in herd C may have been underfed as all dry cows were held in one group irrespective of time to expected calving. It appears as if the dry cows in herds B and D were in a more favourable energy balance ap than the cows in herds A and C. One reason could be that close-up cows in herds B and D were held in a separate group, which facilitated the access to feed. Whitaker [11] suggests a pp reference value for NEFA of <0.7 mmol/L day 10–20 pp. According to this reference value, herds A, C and E all had more than one third of the cows with elevated levels of NEFA the first weeks after calving, indicating an exaggerated mobilisation of adipose tissue also seen as BCS losses. In herd D, few cows were sampled during the period suggested by Whitaker [11] but two more cows had NEFA values above 0.7 mmol/L on day 21. However, despite high BCS ap and pronounced losses in BCS, herd B had normal levels of NEFA pp. Leblanc et al. [14] suggested that elevated NEFA ap increases the risk of abomasal displacement pp.

No significant differences in mean BHB levels were found among the herds. Oetzel [12] suggested 1.4 mmol/L as a threshold for subclinical ketosis. Herds A and D had mean levels around 1.4 mmol/L 5 weeks pp and this may imply abundance of subclinical ketosis. However BHB does not only emanate from incomplete oxidation of NEFA in the liver but also from butyrate of rumen origin oxidised to BHB in the rumen epithelium [37]. Oetzel [12] suggested using a proportion of cows in a given timeframe with elevated levels to evaluate subclinical ketosis on herd level. Thus, over 10% of a minimum of 12 cows sampled days 5–50 pp with BHB values above 1.4 mmol/L has been used as an indication of subclinical ketosis herd-problems. In our herds, between 9% and 33% of the samples were above 1.4 mmol/L. However, only herd D had a clear indication of a herd problem with subclinical ketosis.

Insulin levels ap in herds D and E, and to a lesser extent also in B and C, were similar to levels found in overfed dry cows in a study by Holtenius et al. [38]. In that study cows with high insulin levels ap had a lower glucose clearance rate pp indicating increased insulin resistance. However, the usefulness of insulin levels ap as markers for insulin resistance needs further study. Differences between herds in NEFA and insulin levels were not reflected in glucose levels. This indicates that glucose may not be a sensitive measure of energy status, probably because glucose is subject to tight homeostatic control as previously concluded by Herdt [18]. No significant differences between herds and no changes in level over time were observed in fructosamine. A weak correlation between blood glucose and fructosamine but no association between total protein and fructosamine were found when glucose and total protein were measured 12–30 days before fructosamine [39]. Fructosamine did not seem to be a sensitive marker for glucose levels in healthy cows where the variation in glucose is limited.

Herd A had significantly lower mean cholesterol values than the other herds 1–3 weeks pp. Approximately one third of the cows in this herd had values below 2.0 mmol/L. Low cholesterol levels the first weeks after calving (<2 mmol/L) have been associated with fatty liver pp [22-24]. However, much of the variation in cholesterol may be explained by dry matter intake [21] such that a lower feed intake leads to lower cholesterol levels. The low cholesterol values together with the elevated NEFA levels ap suggests that fatty liver was contributing to the metabolic disorders in herd A. This herd also had the highest frequency of recorded disease during the 12-month period. Herd D had significantly higher mean values of cholesterol both 1–3 weeks ap and 1–3 weeks pp. According to Janovick Guretzky et al. [21], this may be an indication of a lower degree of adipose tissue mobilisation. However, herd D had elevated levels of NEFA and BHB pp, as well as one of the highest reported incidences of disease, thus indicating a high degree of tissue mobilisation.

Herds A and C had higher haptoglobin levels the first week pp than herds D and E (Fig. 3c). These herds also had elevated NEFA levels ap, supporting that elevated haptoglobin levels pp may be associated with fatty liver as previously suggested [28]. A peak in haptoglobin the days after calving are in accordance with several other studies [[40,41], Nyman AK, Emanuelson U, Holtenius K, Ingvartsen KL, Larsen T and Persson Waller K, unpublished data] and probably due to inflammatory reactions in the reproductive tract. Humblet et al. [40] used 0.15 g/L to separate cows with an acute phase response from healthy cows in the first week after parturition. In agreement with another study, we have chosen 0.5 g/L to allow for the expected haptoglobin increase associated with calving [41], but still 27% of the cows sampled from day 2–21 had levels of haptoglobin exceeding 0.5 g/L. Even though the cows in the study were clinically healthy at sampling and samples collected close to recorded disease were omitted, it is possible that undetected infectious or inflammatory processes may have accounted for some of the haptoglobin responses. More research is needed on the levels and kinetics of haptoglobin in naturally occurring fatty liver. The elevated GD levels detected in all five herds (Table 3) indicated liver cell damage, but the herd-specific profiles did not indicate any consistent relationship between haptoglobin, AST and GD in this study. AST had a low diagnostic value on herd level in this study.

Hygienic problems with silage detected in herds D and E and with grain in herds A and B indicated that harvesting or storage or both were not optimal in these herds. Microbial damage to feedstuffs may reduce nutrient content and palatability. Although mycotoxins were not analysed in this study, potentially toxin-producing species were found in feed from herds A, D and E, which may have contributed to the elevated GD levels and possibly to disease frequencies. Herd D had an increase in GD during weeks 7 to 9 pp accompanied by concurrent rises in NEFA and BHB and a decrease in insulin and glucose levels. Herd D changed to the batch of silage (according to the questionnaire) in which hygiene quality problems were detected approximately one week before these blood samples were collected and this may be an explanation for the changes in the blood profile.

The herds were included in the study based on the referring veterinarian's opinion and herd records on disease incidence. In herds B and C, the reported incidence according to SOMRS of DA and ketosis during the studied 12-month period, was lower than the stated inclusion criteria of 6%. However, all herds had a long-term high incidence of DA and had a reported incidence above the Swedish average of abomasal displacement (1.0%) and herds A, B and C had reported incidences above the average for ketosis (1.3%) [29]. The herds were thus judged to be high-incidence herds with respect to Swedish conditions at time of inclusion in the study, but not necessarily in an international perspective.

Establishing reference values for dairy cows in the correct phase of lactation is a challenge. Due to great variations between methods, laboratories and cow material, reference values in literature vary. In order for the present study to be useful for other than Swedish conditions reference values need to be well established. This has led us to use different references for the parameters.

Blood profile results depend on time at sampling in relation to calving, time of day at sampling and the individual cows tested. To get a representative metabolic profile at the herd level, sampling of between 12 and 17 cows is recommended with cows divided into subgroups ap and pp [11,12]. A sufficient number of cows to sample in a narrow time period around calving may only be available in large herds, limiting the usefulness of metabolic profiles in smaller herds. The study herds were sampled when the farmers had time, at or after the morning milking or in the early afternoon. This may have added to variations in blood parameters, but the comparison among herds has not likely been biased because most of the sampling was carried out between 10 and 12 am and sampling time was not systematically different for any of the herds.

We chose to model all blood parameters individually. It is, however, likely that parameters co-vary because they are to some extent related to the same biological processes. It is therefore also possible that a combination of parameters is more useful than each parameter separately. A multivariable approach to the statistical modelling may thus be advantageous and should be addressed in future research.

## Conclusion

In all herds, dry cows were overconditioned and showed substantial losses in body condition during the first 4–6 weeks pp. NEFA was the parameter that most closely reflected the BCS losses, supporting earlier findings of its usefulness in diagnosing herd problems. The BCS losses were not reflected in glucose and fructosamine levels. One herd differed in insulin and cholesterol patterns suggesting that these parameters may be potentially useful in herd profiles, but this needs further investigation. Increased GD suggested liver cell damage in all herds.

## Competing interests

The authors declare that they have no competing interests.

## Authors' contributions

The study was designed by all authors and LS did the field work and collected the data. The statistical analysis was carried out by LS and UE. All authors contributed to the interpretation of the data. LS drafted the manuscript and all authors revised and finally read and approved the presented manuscript.

## References

[B1] Hayirli A, Grummer RR, Nordheim EV, Crump PM (2002). Animal and dietary factors affecting feed intake during the prefresh transition period in Holsteins. J Dairy Sci.

[B2] Rukkwamsuk T, Kruip TA, Wensing T (1999). Relationship between overfeeding and overconditioning in the dry period and the problems of high producing dairy cows during the postparturient period. Vet Q.

[B3] Goff JP, Horst RL (1997). Physiological changes at parturition and their relationship to metabolic disorders 1, 2. J Dairy Sci.

[B4] Bobe G, Young JW, Beitz DC (2004). Invited review: Pathology, etiology, prevention, and treatment of fatty liver in dairy cows. J Dairy Sci.

[B5] Ward WR, Murray RD, White AR, Rees EM, Garnsworthy PC, Cole DJA (1995). The use of blood biochemistry for determining the nutritional status of dairy cows. The Annual Nutrition Conference for Feed Manufacturers; University of Nottingham.

[B6] Payne JM, Dew AM, Manston R, Faulks M (1970). The use of a metabolic profile test in dairy herds. Vet Rec.

[B7] Kida K (2002). Use of every ten-day criteria for metabolic profile test after calving and dry off in dairy herds. J Vet Med Sci.

[B8] Ingraham RH, Kappel LC (1988). Metabolic profile testing. Vet Clin North Am Food Anim Pract.

[B9] Blowey RW (1975). A practical application of metabolic profiles. Vet Rec.

[B10] Mulligan FJ, O'Grady L, Rice DA, Doherty ML (2006). A herd health approach to dairy cow nutrition and production diseases of the transition cow. Anim Reprod Sci.

[B11] Whitaker DA, Andrews AH, Blowey RW, Boyd H, Eddy RG (2004). Metabolic profiles. Bovine Medicine: Diseases and Husbandry of Cattle.

[B12] Oetzel GR (2004). Monitoring and testing dairy herds for metabolic disease. Vet Clin North Am Food Anim Pract.

[B13] Macrae AI, Whitaker DA, Burrough E, Dowell A, Kelly JM (2006). Use of metabolic profiles for the assessment of dietary adequacy in UK dairy herds. Vet Rec.

[B14] LeBlanc SJ, Leslie KE, Duffield TF (2005). Metabolic predictors of displaced abomasum in dairy cattle. J Dairy Sci.

[B15] Geishauser T, Leslie KE, Duffield TF, Edge V (1997). Evaluation of aspartate transaminase activity and beta-hydroxybutyrate concentration in blood as tests for prediction of left displaced abomasum in dairy cows. Am J Vet Res.

[B16] Ropstad E (1987). Serum fructosamine levels in dairy cows related to metabolic status in early lactation. Acta Vet Scand.

[B17] Jensen AL, Petersen MB, Houe H (1993). Determination of the fructosamine concentration in bovine serum samples. J Vet Med A Physiol Pathol Clin Med.

[B18] Herdt TH (2000). Variability characteristics and test selection in herd-level nutritional and metabolic profile testing. Vet Clin North Am Food Anim Pract.

[B19] Martin GJ, Rand JS (2007). Comparisons of different measurements for monitoring diabetic cats treated with porcine insulin zinc suspension. Vet Rec.

[B20] Davison LJ, Herrtage ME, Catchpole B (2005). Study of 253 dogs in the United Kingdom with diabetes mellitus. Vet Rec.

[B21] Janovick Guretzky NA, Carlson DB, Garrett JE, Drackley JK (2006). Lipid metabolite profiles and milk production for Holstein and Jersey cows fed rumen-protected choline during the periparturient period. J Dairy Sci.

[B22] Top AM Van den, Van Tol A, Jansen H, Geelen MJH, Beynen AC (2005). Fatty liver in dairy cows *post partum *is associated with decreased concentration of plasma triacylglycerols and decreased activity of lipoprotein lipase in adipocytes. J Dairy Res.

[B23] Steen A, Grönstöl H, Torjesen PA (1997). Glucose and insulin responses to glucagon injection in dairy cows with ketosis and fatty liver. J Vet Med A Physiol Pathol Clin Med.

[B24] Holtenius P, Niskanen R, Holtenius K, Hartigan PJ, Monaghan ML (1986). Blood lipids and lipoproteins in cows with abomasal displacement. 14 World Buiatrics Congress; Dublin.

[B25] Kaneko JJ, Harvey JW, Bruss ML (1997). Clinical Biochemistry of Domestic Animals.

[B26] Alsemgeest SPM, Kalsbeek HC, Wensing T, Koeman JP, Van Ederen AM, Gruys E (1994). Concentrations of serum amyloid-A (SAA) and haptoglobin (HP) as parameters of inflammatory diseases in cattle. Vet Q.

[B27] Yoshino K, Katoh N, Takahashi K, Yuasa A (1992). Purification of a protein from serum of cattle with hepatic lipidosis and identification of the protein as haptoglobin. Am J Vet Res.

[B28] Katoh N, Nakagawa H (1999). Detection of haptoglobin in the high-density lipoprotein and the very high-density lipoprotein fractions from sera of calves with experimental pneumonia and cows with naturally occurring fatty liver. J Vet Med Sci.

[B29] Swedish Dairy Association (2006). Animal health 2005/2006: Annual report from the animal health section Djurhälsovård 2005/2006 Djurhälsa: Redogörelse för Husdjursorganisationens Djurhälsovård (in Swedish).

[B30] Edmonson AJ, Lean IJ, Weaver LD, Farver T, Webster G (1989). A body condition scoring chart for Holstein dairy cows. J Dairy Sci.

[B31] Dohoo IR, Martin W, Stryhn H (2003). Veterinary Epidemiologic Research.

[B32] Chełkowski J (1991). Cereal grain mycotoxins, fungi and quality in drying and storage Developments in food science, 26.

[B33] Swedish Board of Agriculture (2006). Directions and general recommendations concerning feed SJVFS 2006:81 Saknr M 39 Allmänna råd till 4 kapitlet 6§(in Swedish).

[B34] Morrow DA (1976). Fat cow syndrome. J Dairy Sci.

[B35] Duffield TF (2000). Subclinical ketosis in lactating dairy cattle. Vet Clin North Am Food Anim Pract.

[B36] Odensten MO, Chilliard Y, Holtenius K (2005). Effects of two different feeding strategies during dry-off on metabolism in high-yielding dairy cows. J Dairy Sci.

[B37] Kristensen NB, Gäbel G, Pierzynowski SG, Danfær A (2000). Portal recovery of short-chain fatty acids infused into the temporarily-isolated and washed reticulo-rumen of sheep. Br J Nutr.

[B38] Holtenius K, Agenas S, Delavaud C, Chilliard Y (2003). Effects of feeding intensity during the dry period. 2. Metabolic and hormonal responses. J Dairy Sci.

[B39] Tråvén M, Stengärde L, Tråvén M, Emanuelson U, Holtenius K, Hultgren J, Niskanen R, Fürll M (2007). Evaluation of fructosamine as a tool to assess glucose levels in dairy cows. 13th International Conference on Production Diseases in Farm Animals; Leipzig.

[B40] Humblet MF, Guyot H, Boudry B, Mbayahi F, Hanzen C, Rollin F, Goudeau JM (2006). Relationship between haptoglobin, serum amyloid A and clinical status in a survey of dairy herds during a 6-month period. Vet Clin Pathol.

[B41] Uchida E, Katoh N, Takahashi K (1993). Apperance of haptoglobin in serum from cows at parturition. J Vet Med Sci.

